# Expedited referrals from community health center to opioid treatment program: innovative approaches to improving access to methadone treatment for patients who use opioids and experience homelessness

**DOI:** 10.1186/s13722-025-00627-1

**Published:** 2025-12-02

**Authors:** Natalie Stahl, Amy Bositis, Carolyn Damato-MacPherson, Rebecca Weiner, Dianna Conole, Ann Scheck McAlearney, Henry M. Stadler, Avik Chatterjee

**Affiliations:** 1 Greater Lawrence Family Health Center, Lawrence, MA USA; 2 Boston Medical Center, Boston, MA USA; 3 Lawrence Comprehensive Treatment Center, Lawrence, MA USA; 4 Department of Family and Community Medicine, College of Medicine, The Ohio State University, Columbus, OH USA; 5 Section of General Internal Medicine, Department of Medicine, Boston University Chobanian & Avedisian School of Medicine, Boston, MA USA

**Keywords:** Opioid, Methadone, Homelessness

## Abstract

**Background:**

Methadone treatment (MT) is the “gold standard” treatment for opioid use disorder (OUD). However, patients face significant barriers to enrollment at opioid treatment programs (OTPs), e.g. need for proof of identity, lack of transportation, and limited intake hours. For patients experiencing homelessness or unstable housing, those barriers are magnified.

**Objective:**

We created a multifactorial intervention to address the above-described barriers to facilitate prompt OTP admission for patients with OUD and housing instability.

**Methods:**

Our target population was patients with OUD and unstable housing who utilize the Greater Lawrence Family Health Center mobile health unit (MHU) and syringe services program (SSP) in Lawrence, MA. As part of the HEALing Communities Study, we developed an expedited referral process whereby mobile health clinicians provided preliminary clearance for MT. SSP staff, recovery coaches, and OTP staff assisted with outreach, engagement, coordination, and intake. Patients who completed psychosocial intake could start dosing at the OTP within 1–3 days.

**Results:**

Over six months, 87 individuals were linked to treatment and 64 were admitted and dosed at the OTP. Many patients who had previously assumed they could not seek treatment with methadone were eager to do so when barriers to access were reduced.

**Conclusions:**

Efforts to reduce barriers to methadone initiation via partnerships between mobile health programs, SSPs, and OTPs can be a tool in combating the overdose crisis.

## Introduction

Opioid overdose deaths continue to increase both nationally and in Massachusetts and disproportionately affect people experiencing homelessness and communities of color [1, 2]. Methadone treatment (MT) is the “gold standard” treatment for opioid use disorder (OUD). As fentanyl has overtaken the illicit opioid supply, more patients are having difficulty with initiation and/or continuation of buprenorphine [3], making access to MT even more critical. However, the structural and systemic barriers to MT enrollments - barriers which disproportionately affect people experiencing homelessness (PEH) - make MT enrollment unnecessarily difficult. Overcoming those barriers requires community-specific interventions that address the local disease burden and gaps in community resources.

Lawrence, Massachusetts is a former mill town located 30 miles north of Boston at the intersection of two major highways in the Northeast. The community identifies as primarily Hispanic (76%) mostly from the Dominican Republic and Puerto Rico [4]. Lawrence’s proximity to highway conduits for the movement of illicit substances has resulted in higher quantities and lower prices of fentanyl compared to in surrounding communities [5] and continues to drive the OD fatality rate higher than in other Massachusetts cities [6]. Greater Lawrence Family Health Center (hereafter referred to as the Health Center) is a federally qualified health center (FQHC) that serves 58,000 patients in the Merrimack Valley of Massachusetts. The Health Center has seven “brick and mortar” clinics in the region, two school-based health centers, and a Healthcare for the Homeless Program that provides health care to 750 patients in local shelters, drop-in centers and on two mobile health units (MHUs). In addition to the full spectrum of primary care, the Health Center serves 500 people living with addiction through the Office-Based Addiction Treatment (OBAT) program, of which about 200 prefer to seek care at a MHU. The Health Center also operates a community-based services center that offers syringe services programs (SSPs), confidential HIV and STI testing, naloxone distribution, recovery coaching, and other services.

Lawrence Comprehensive Treatment Center (hereafter referred to as the OTP) is one of three OTPs in the greater Lawrence area and is about a 5-minute walk from the community-based services center SSP and bridge clinic, making it an ideal partner to provide OTP-level services to this population. The OTP has been operating in the community for 25 years and currently doses approximately 650 people each month. The purpose of this intervention was to reduce barriers to methadone initiation by making same-day or next-day OTP enrollment available to PEH via a collaboration between the Health Center bridge clinic and a nearby OTP. The intervention period was from July 1 to December 31, 2023. In this Short Communication we describe this FQHC-based expedited referral program for MT that sought to provide access to same- or next-day methadone initiation for marginalized individuals with OUD.

## Methods

As part of the HEALing (Helping to End Addiction Long-term) Communities Study (HCS), a multi-site study in 67 communities across four states (Ohio, Massachusetts, Kentucky, and New York) that used the Communities that HEAL (CTH) intervention with the aim of reducing opioid overdose deaths, the Health Center and the OTP identified specific challenges patients with opioid use disorder in Lawrence faced when trying to access methadone treatment.

Some of the barriers identified included: being unable to get to OTP due to distance or lack of transportation, needing formal proof of identity, and limited OTP intake options (i.e., only available once per week, needing to come early morning, needing to attend multiple separate appointments to complete paperwork). For patients experiencing homelessness or unstable housing - many of whom lack photo identification, cell phones, and/or reliable transportation - those barriers are magnified.

As part of the CTH intervention, the Health Center and the OTP expanded access hours to establish a Bridge Clinic and collaborated to develop an expedited referral process for initiation of MT. The Health Center added 20 new hours of afternoon services on the MHU in the parking lot of the Health Center adjacent to the SSP. These services included primary care, addiction treatment, infectious disease treatment and recovery coaching support. These MHU sessions comprised the Health Center’s new Bridge Clinic program.

The expedited MT referral process included: (1) OTP accepting preliminary medical evaluation from Health Center physicians; (2) OTP stationing a behavioral health clinician at the Bridge Clinic to complete psychosocial assessment and other intake paperwork; and (3) OTP (with support from the State Opioid Treatment Authority) accepting a Health Center EMR face-sheet as a temporary ID. Recovery coaches (two in total) facilitated this entire process, describing the possibility of expedited MT referral during street and SSP outreach, greeting patients at the Bridge clinic and connecting them to Health Center physicians and OTP behavioral health clinicians, and acquiring temporary IDs, cell phones, applying for state Medicaid transportation if needed, and addressing other logistical barriers.

## Results

**Fig. 1:**
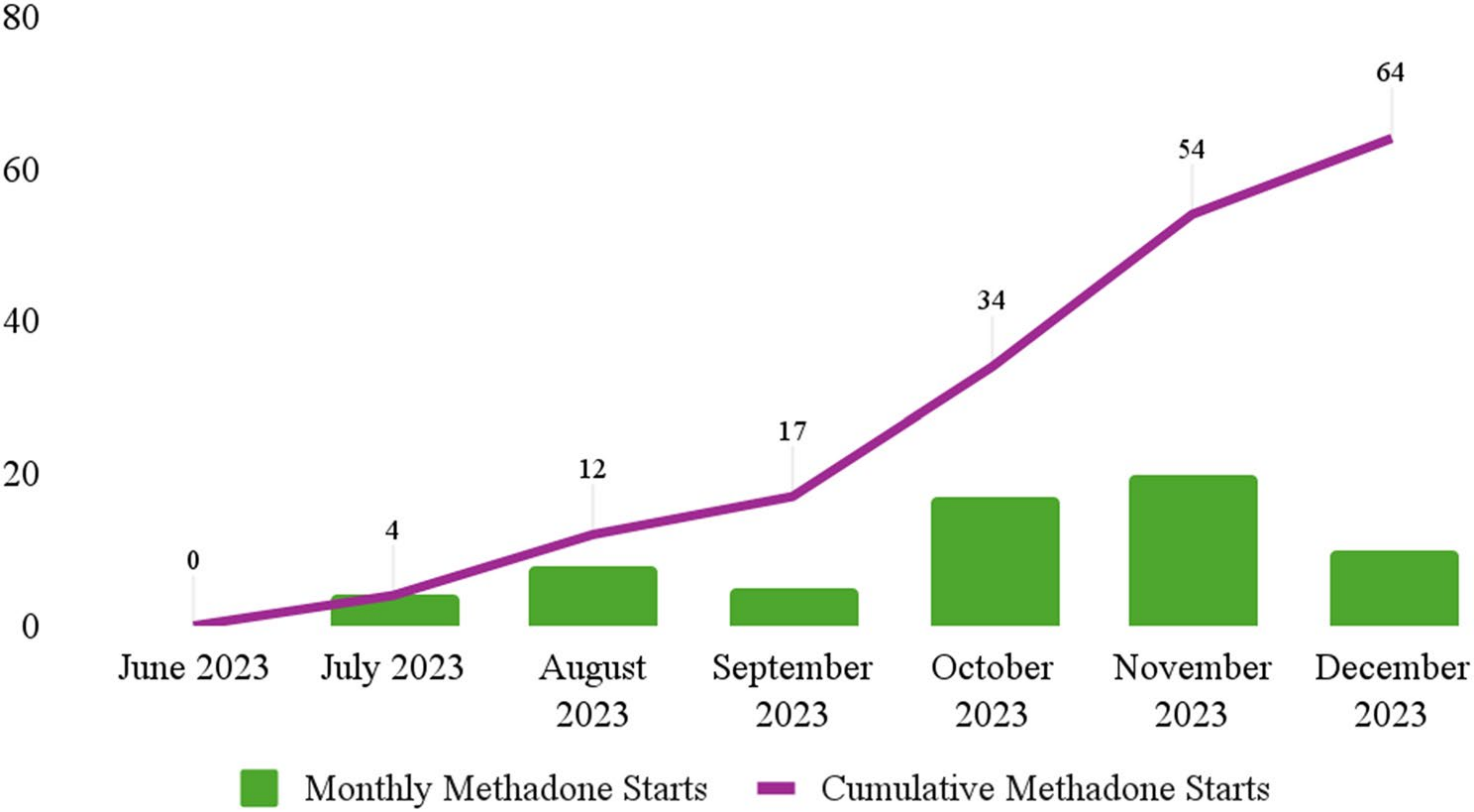
The monthly and cumulative number of patients started on methadone via the expedited referral process for the duration of the study intervention

Through the expedited referral process, 87 individuals were linked to the OTP for psychosocial evaluations in the 6 months of data collection. Of the individuals linked, 64 were admitted and dosed (Fig. 1). Individuals were 56% male, 27% from ages 18–34, 48%, from ages 35–54, 39% Hispanic/Latinx, and 53% non-Hispanic white. By implementing the expedited referral process between the Health Center and OTP, several barriers to MT were reduced or eliminated (Table 1). Most notably, the partnership between the OTP and Health Center and co-location of services provided a continuum of care for individuals needing treatment. Patients who had not sought MT before the intervention period could successfully start. No adverse/unintended events were observed.

**Table 1:** Identified gaps in care preventing access to methadone for patients in Lawrence, MA and the strategies implemented to mitigate them

Gap	Strategy	Successes	Ongoing or Anticipated Challenges
**Identification:** *“IDs have been a huge obstacle.”*	Face-sheet from FQHC	Patients who otherwise would not have been able to initiate methadone due to lack of identification were able to start using their face-sheet.	Lack of official identification is still a barrier to accessing other resources.
**Time to First Dose:** Lack of OTP staff capacity, and limited OTP hours of operation impeded OTP ability to start patients on MOUD.	Medical Evaluation from FQHC OTP counselor presence in afternoons to complete paperwork	During the month of October, 10 patients received an initial dose the next business day.	Financial sustainability of staffing expanded OTP counselor hours for biopsychosocial intake.
**Continuity of Care:** Limited communication between OTP and FQHC	Colocation of OTP and FQHC staff on MHU Interagency cooperation between OTP and FQHC	Sustained close, collaborative relationship among staff from both agencies. Shared goals and belief in the mission sustained beyond the study.	(1) Limited access to MHU depending on weather (i.e., limited waiting area). (2) Anticipated challenges that come along with staffing changes.
**Health Related Social Needs:** Lack of access to transportation/housing was a barrier to engage with social services and substance use treatment.	Recovery Coaching and wraparound services (e.g. (identification, social security cards, transportation, housing, and phones)	*“Recovery Navigator was able to obtain 3 birth certificates for clients; also able to successfully apply for 4 government smart phones; assisted one client with disability claim; assisted several clients with information pertaining to obtaining MA IDs and licenses; arranged to accompany 2 clients to the RMV to get their MA IDs. It has been very helpful for this RN to split time between Lawrence CTC and GLFHC - to continually meet with new people and inform them about the Methadone clinic.”*	The cost of vital document re/production and transportation are a barrier. Stringent rules for Medicaid funded transportation make it inaccessible to the patient population. Navigating government systems for benefits is time consuming and confusing. The biggest issue facing clients is housing, which study funds could not be used towards. *“There have been major roadblocks regarding assisting clients with housing needs. This recovery navigators and others at Lawrence CTC have referred clients to [housing coordinator], only to find that she is never available to meet with them, nor does she ever return phone calls, including those from this RN [Recovery Navigator]. This is an ongoing frustration.”* *“The homeless population has been quite a challenge now that the cold weather is upon us. We consistently try to connect our clients with the new housing funding for the rooming houses with the Lawrence Housing Authority, but our clients are returning with the same stories, they are promised entrance into these rooming homes, but it never actually transpires.”*

## Discussion

The expedited referral process allowed patients to complete OTP intake with minimal barriers and outside the traditional hours of the OTP, and in most cases, receive their first dose of methadone within 24 hours. Patients who might have assumed they could not access methadone or might have struggled to complete the traditional intake process, were thus able to access evidence-based life-saving treatment. While we did not collect historical data on connections to the OTP from the SSP (since most referrals were informal and involved patients themselves calling to make appointments), the substantial number of documented OTP connections after implementation suggest that the intervention was an improvement to the referral process.

Many of the strategies to reduce barriers to methadone themselves incur no institutional financial cost; most notably, assuming a medical visit can be billed and reimbursed, there is no significant cost to providing medical evaluation for methadone or printing out a clinic face-sheet for identification. The salaries of the Health Center recovery coach on the MHU Bridge Clinic who supports clients in accessing medications for opioid use disorder (MOUD) and the cost of the OTP behavioral health provider completing the psycho-social intake outside of typical OTP hours are notable costs, particularly since these positions are critical but do not generate revenue.

After the HCS intervention period ended, both the Health Center and OTP secured grant funding through the Massachusetts Bureau of Substance Abuse Services to sustain these positions. The number of patients seeking methadone initiation via expedited referral process has stabilized to about one per week which raises the question of long-term sustainability without grant funding. However, recent changes to OTP regulations (42 CFR part 8 rule) – including, for example, allowing OTP psycho-social evaluations to be completed via telehealth, and allowing OTPs to utilize physical examinations by non-OTP practitioners in some circumstances - provide multiple options for adjusting expedited referral processes into the future [7]. Moreover, the Health Center is finalizing plans to administer one to three days of methadone under the “3-day rule” (21 CFR 1306.07(b)) which would provide additional avenues for same-day methadone initiations [8].

This intervention was largely able to circumvent transportation barriers (though not housing barriers), because the SSP, Bridge Clinic, OTP, and homeless encampments where many patients lived were within walking distance, but transportation barriers and lack of housing are pervasive barriers for engagement in addiction treatment for many individuals. The syndemic of homelessness and the opioid overdose crisis is increasingly at the forefront of public discourse, both because of the public health burden it represents as well as the highly visible challenges it places on local governments. Unstable housing or homelessness is prevalent among individuals with OUD, and overdose is a leading cause of death among PEH; in one recent cohort study of patients experiencing homelessness in Boston, overdose accounted for one in four deaths [9]. To address this syndemic, it is critical that access to all evidence-based treatments for OUD (including methadone) be available to PEH. Too often, the “failure” of PEH to access or continue treatment for OUD is ascribed to personal characteristics such as lack of motivation. These interventions could include explicit policies allowing for interim forms of identification (from medical providers, criminal legal system, etc.) to be used; open-access OTP enrollment (i.e., 5 days/week); expedited referrals between primary care providers and OTPs (i.e., allowing physical examinations and OUD diagnoses to provide medical clearance for enrollment); and collaborations between OTPs and harm reduction programs. Successful public health interventions are grounded in the philosophy that we must make “the healthy choice the easy choice” [10, 11] - and the same principle should be applied to the homelessness/OUD syndemic. Structural and public health interventions to increase access to methadone are evidence-based and feasible, and should be supported by State Opioid Treatment Authorities, OTP directors, primary care providers, harm reduction advocates, and funders.

## Limitations

A limitation of this analysis is that we did not have a formal comparison group, a comparator site, or historical comparator data. Historically, there were no same-day handoffs between the FQHC and the OTP. Secondly, while we sought to address barriers to methadone initiation with this project, we did not address retention on methadone treatment, which is also vitally important and subject to numerous barriers. Additionally, while we present some ongoing implementation challenges in Table 1, formal and ongoing implementation feedback will be important. As noted in the Table, lack of housing remained a barrier that was beyond the scope of this intervention to address, and while a state Medicaid transportation program exists, complex rules and requirements to access it remain a barrier. Finally, the findings from this study are derived from a single site in Lawrence, MA, a state which has historically been supportive of expanding substance use treatment programming. As such, the findings may not be generalizable to other states.

## Conclusions

We describe the feasibility and efficacy of an expedited referral protocol via collaboration between an FQHC and OTP in Lawrence, MA. This success of this intervention suggests that efforts to reduce barriers to methadone initiation via partnerships between mobile health programs, SSPs, and OTPs can be a tool in combating the overdose crisis by improving access to MT for PEH with OUD.

## Data Availability

No datasets were generated or analysed during the current study.
